# Quantifying late gadolinium enhancement on CMR provides additional prognostic information in early risk-stratification of nonischemic cardiomyopathy: a cohort study

**DOI:** 10.1186/1471-2261-14-110

**Published:** 2014-08-27

**Authors:** Pauli Pöyhönen, Sari Kivistö, Miia Holmström, Helena Hänninen

**Affiliations:** Heart and Lung Center, Division of Cardiology, Helsinki University Central Hospital, Po BOX 340, 00029 HUCH Helsinki, Finland; Medical Imaging Center, Helsinki University Central Hospital, Helsinki, Finland

**Keywords:** Cardiomyopathy, Nonischemic cardiomyopathy, Cardiovascular magnetic resonance imaging, Late gadolinium enhancement, Segmental wall motion abnormality, Prognosis, Risk-stratification

## Abstract

**Background:**

Suspected nonischemic cardiomyopathy (NICM) is a common clinical setting with highly variable prognosis. Early noninvasive risk-stratification is important for justification of invasive examinations, specific treatment and patient surveillance. We studied the additional prognostic value of late gadolinium enhancement (LGE) and segmental wall motion abnormality (SWMA) extent on cardiovascular magnetic resonance (CMR) compared to traditional risk factors in suspected NICM.

**Methods:**

In this observational cohort study, we enrolled 86 consecutive patients referred for CMR due to suspected NICM. Patients with ischemic cardiomyopathy were excluded. CMR images were analysed for left ventricular LGE and SWMA extents and patients were followed-up for major adverse cardiac events (MACE), including cardiovascular death, aborted sudden death and cardiac transplantation.

**Results:**

Of 86 patients (median age: 53 years, 45% female), mainly presenting with ventricular arrhythmias (40%) and congestive heart failure (44%), 76% were finally diagnosed with NICM, 17% with left ventricle hypertrophy and 7% with idiopathic arrhythmia. On CMR, 61 patients (71%) had LGE and 56 (65%) SWMA. During median follow-up of 835 days, 15 patients (17%) reached MACE. In univariant analysis, LGE volume (hazard ratio [HR] 1.028 per 1% increase in LGE, p < 0.001), left ventricular ejection fraction (LVEF) (HR 0.959, p = 0.009) and SWMA score (HR 1.067, p = 0.012) had strongest associations with MACE. In multivariate analysis, the best overall model for event prediction included LGE volume (HR 1.027, p = 0.003), sustained ventricular tachycardia (HR 4.7, p = 0.011) and LVEF (HR 0.962, p = 0.034). Among patients with LGE, there was an event rate of 26% (14 of 61) versus 4% (1 of 25) in patients without LGE (p = 0.041, Log-rank). The highest event rate was observed in patients with LGE volume of ≥17%. Patients without SWMA did not experience MACE (p = 0.002, Log-rank), giving additional information in the subgroup of patients with preserved LVEF (≥50%).

**Conclusions:**

In suspected NICM, presenting with ventricular arrhythmias or heart failure, LGE extent gives additional prognostic information compared to traditional risk factors, while the absence of SWMA may give prognostic information beyond normal LVEF. Even though the final diagnosis is uncertain in NICM, extensive amount of LGE should be considered as a sign of poor prognosis.

## Background

Nonischemic cardiomyopathy (NICM) is a common diagnostic challenge in clinical cardiology. NICMs are a diverse group of myocardial diseases associated with mechanical or electrical dysfunction and usually inappropriate ventricular hypertrophy or dilatation, not related to atherosclerosis [1]. The etiology of NICM may be genetic, acquired or secondary to systemic disease, with highly variable clinical presentation and prognosis [2, 3]. However, the specific diagnosis of NICM is initially often unexplained and reaching the final diagnosis may be a timely process. Thus, early risk-stratification of NICM is valuable for justification of potentially harmful invasive examinations [4] and proper patient surveillance. Although left ventricular ejection fraction (LVEF), ventricular tachycardia (VT) and NYHA functional classification are all well known predictors of cardiac mortality [5], there is increasing evidence that noninvasive cardiovascular magnetic resonance (CMR) imaging with late gadolinium enhancement (LGE) provides additional information in the risk-stratification of NICM [6–9].

The prognostic value of LGE has been shown in several specific cardiac diseases, including ischemic and nonischemic cardiomyopathies [10–15]. LGE, i.e. delayed enhancement on CMR images after intravenous injection of gadolinium-contrast, visualizes increases in the regional extracellular space related to myocardial necrosis, fibrosis, oedema or infiltration. Ideally, the prognostic value of LGE should be interpreted in the context of the specific disease, since the etiology of NICM itself carries a prognostic value [3]. However, since the specific diagnosis of NICM often remains unclear even after CMR, the prognostic value of LGE must frequently be considered in suspected or newly diagnosed NICM. There is limited information on the prognostic value of LGE in nonselected consecutive patients with newly diagnosed NICM, which reflects the usual real-life clinical scenario [8]. Furthermore, most studies have evaluated the prognostic value of the presence of LGE [9], and there are only few studies on quantifying the extent of LGE in NICM, considering the best prognostic ability [10, 16, 17].

In this study, the visual scoring method based on the standard 17-segment model was used to estimate the global extent of LGE in the left ventricle (LV) [18]. This method has been shown to be rapid and accurate method to estimate LGE both in ischemic and nonischemic cardiomyopathies [7, 19, 20].

Segmental wall motion abnormalities (SWMA), visualized by echocardiography, have been used for long to detect myocardial ischemia and viability in ischemic cardiomyopathy [21]. However, CMR with standard cine-imaging has been shown to be the most accurate and reproducible method to study left ventricular regional function [22, 23]. Recently, visual scoring of SWMA has been shown to be accurate and reproducible method to estimate LVEF, known strong predictor of cardiac mortality [24]. Nevertheless, it is unclear if SWMA gives additional prognostic information beyond LVEF in NICM.

In this cohort study, we evaluated the additional prognostic value of LGE and SWMA extent on CMR compared to traditional cardiac risk factors in a common clinical setting of suspected NICM. We hypothesized that even though the eventual diagnosis is uncertain in suspected NICM, LGE or SWMA extent might provide valuable information in early risk-stratification.

## Methods

### Study design and patient selection

This observational cohort study was performed in the Heart and Lung Center at the Helsinki University Central Hospital (HUCH). Altogether 98 consecutive patients referred for LGE CMR due to suspected NICM between November 2008 and April 2010 (18 months) were enrolled to the study. All enrolled patients had suspected NICM, i.e. symptoms of heart failure, mechanical and/or electrical cardiac dysfunction, usually associated with inappropriate ventricular dilatation or hypertrophy, or cardiac enzyme elevation, not related to atherosclerosis [1]. Before CMR, no patient had history of myocardial infarction or documented significant coronary artery disease (CAD) defined as > 50% stenosis in two or more epicardial vessels or > 50% stenosis in left main or proximal left anterior descending artery [25]. Also, no patient had known valvular or congenital heart disease. After CMR, six patients were lost due to lack of complete baseline data and another six patients were excluded due to ischemic cardiomyopathy. Thus, eventually 86 patients were eligible for analysis. The study protocol was approved by the institutional review board of HUCH. All data in this study were analysed retrospectively.

### Data acquisition

Medical records were reviewed for traditional cardiovascular prognostic factors: patient demographics, cardiovascular risk factors, cardiac symptoms and clinical signs. Patients underwent extensive cardiac evaluation for underlying diagnosis: CMR (n = 86, 100% of patients), echocardiography (n = 86, 100%), coronary angiography (n = 47, 55%), stress and rest single-photon emission computed tomography (SPECT) (n = 4, 5%), one or more endomyocardial biopsies (n = 41, 48%; total number of biopsy procedures 55), positron emission tomography (PET) (n = 21, 24%), electrophysiological study (n = 14, 16%), mediastinoscopy (n = 4, 5%) and explant histopathology after heart transplantation (n = 2, 2%). The final diagnosis was reached using all available clinical information following the AHA 2006 guidelines of classification of cardiomyopathies [1].

Coronary angiography was done predominantly before CMR, based on the decision of treating cardiologist. Patients examined with neither angiography nor SPECT (n = 37, 43%) were relatively young (median age = 45 years), had only few CAD risk factors (the median number of risk factors was 1 out of 5 [dyslipidemia, hypertension, diabetes, smoking, family], interquartile range 0–1) and evident nonischemic etiology for symptoms. Patients were excluded due to ischemic cardiomyopathy if they had documented significant CAD, definition see above, presence of reversible perfusion defect in stress and rest SPECT or presence of CAD in explanted hearts.

Nonsustained VT was defined as six or more consecutive ventricular complexes lasting less than 30 seconds, and sustained VT with a duration of 30 seconds or more. Congestive heart failure (CHF) was defined using the 2008 guidelines of the European Society of Cardiology with the requirement of objective sign of fluid retention.

### CMR protocol and image analysis

CMR imaging was performed with a 1.5-T imager (Avanto; Siemens, Erlangen, Germany) using 12-channel body-array coil as a receiver. Breath-hold cine CMR was performed using retrospectively electrocardiographically gated segmented true fast imaging with steady-state free-precession (SSFP). Cine CMR images were acquired in vertical, horizontal long-axis and short-axis planes covering the whole left ventricle. Typical imaging parameters were TR/TE 3.0/1.6 ms, flip angle 52 degrees, 256 x 256 matrix and 240 x 340 mm field of view. Slice thickness was 6 mm and interslice gap 100% (6 mm). The temporal resolution was 42 – 49 ms. Five to fifteen minutes after intravenous injection of a contrast agent (gadoterate meglumine, Dotarem® 0.1 mmol/kg) LGE images were acquired in the same views as for cine images, using inversion-recovery turbo fast-low angle shot (FLASH). Typical imaging parameters were TR/TE 2.58/2.3 ms, flip angle 50 degrees, 256 x 256 matrix, and 240 × 340 mm field of view. Slice thickness was 8 mm and interslice gap 100% (8 mm). Inversion times were optimized to null the signal intensity of normal myocardium (240 – 360 ms).

CMR images were analysed for the presence and extent of left ventricular LGE and SWMA by experienced cardiac radiologist blinded to clinical outcome. LGE was evaluated using the previously described visual scoring method based on the standard 17-segment model of the left ventricle [18]. In each segment, the percentage of enhancement was visually estimated and scored as 0 (no enhancement), 1 (0 – 25% enhancement), 2 (26 – 50% enhancement), 3 (51 – 75% enhancement) or 4 (76 – 100% enhancement). The global extent of LGE (LGE score) was calculated summing all segmental scores. To estimate the volume of LGE in the left ventricle, LGE score was then expressed as a percentage of the total maximum score (4 × 17 = 68) using formula: 100 × (LGE score) / 68.

Similarly, SWMA was visually estimated based on the 17-segment model using the scoring method validated for echocardiography and later used in CMR studies [21]. The degree of wall motion abnormality in each segment was scored as 0 (normokinesia), 1 (hypokinesia), 2 (akinesia) or 3 (dyskinesia). The global SWMA score of the left ventricle was then calculated as the sum of all segmental scores.

### Patient follow-up and endpoints of the study

After CMR, patients were followed-up for major adverse cardiac events (MACE), including cardiovascular death, aborted sudden death or cardiac transplantation until April 30th 2012, based on information from medical records and mortality data from the national registry of Statistics Finland. For MACEs, event times were measured from the time of CMR to the first event. Aborted sudden death was defined as documented resuscitation from cardiac arrest or appropriate implantable cardioverter-defibrillator therapy, i.e. antitachycardia pacing or shock, for VT or ventricular fibrillation (VF). To meet the endpoint criteria, an event had to be distinct from baseline arrhythmias.

### Statistical analysis

Continuous variables are presented as median (interquartile range [IQR]) and categorical variables as frequency (%), unless otherwise mentioned. Comparison between continuous variables was performed with Mann–Whitney U test and between categorical variables with Pearson Chi-Square test with continuity correction, Fisher’s exact test or Mann–Whitney U test. A p-value of < 0.05 was considered statistically significant and all statistical tests were 2-sided. Univariate Cox regression analysis was performed to study the prognostic significance of each predictive factor separately. Variables with statistical significance p < 0.05 (entry cut-off) were considered in the multivariate model. Forward stepwise multivariate Cox regression analysis was performed to study the independency of predictive variables, with a removal cut-off value of p = 0.05 for the final model. The number of variables in the model was limited to three in accordance with the limited number of events during follow-up (at least 5 events per each covariate). All variables in the multivariate model were tested to satisfy Cox proportional hazard assumption by plotting hazard function and logarithm of hazard function. If needed, continuous variables were made to dichotomous and cut-off values were taken from literature or close to median. Kaplan-Meier method was used to plot and compare (Log rank) survival curves. Receiver operating characteristic (ROC) curves were used to find the best cut-off values of LGE extent and SWMA extent (optimal combination of sensitivity and specificity) for the prediction of events. Statistical analysis was performed on SPSS 20 statistical package (SPSS, Chigaco, IL).

## Results

### Patient characteristics

Of the 86 patients with suspected NICM the median age was 53 (42 – 61) years at the time of CMR and 39 (45%) were female. Most common symptoms at presentation were decline in functional capacity (67%), ventricular arrhythmias (40%) and CHF (44%). After extensive cardiac examinations, of all cohort patients suspected for NICM at baseline, 65 patients (76%) were finally diagnosed with NICM, 15 (17%) with left ventricle hypertrophy and 6 (7%) with idiopathic arrhythmia, see Table 1. The most frequent disease entities were inflammatory cardiomyopathy (n = 23, 27%) and dilated cardiomyopathy (DCM) (n = 22, 26%). If a patient had several concomitant cardiac diseases, the disease considered to cause the current cardiac symptoms is presented.

**Table 1:** **Final diagnoses of all cohort patients suspected for nonischemic cardiomyopathy**

	All patients (n = 86)
Nonischemic cardiomyopathy*	65 (76)
Inflammatory cardiomyopathy	23 (27)
Dilated cardiomyopathy	22 (26)
Cardiomyopathy nonspecific	8 (9)
Infiltrative cardiomyopathy or storage disease	5 (6)
Noncompaction cardiomyopathy	3 (3)
Hypertrophic cardiomyopathy	2 (2)
Tako-Tsubo cardiomyopathy	1 (1)
Ion channelopathy (Long-QT syndrome)	1 (1)
**Other diagnoses**	21 (24)
Left ventricular hypertrophy**	15 (17)
Idiopathic arrhythmia	6 (7)

Values are n (%).

*Classification of (nonischemic) cardiomyopathies based on AHA 2006 guidelines [1]. Cardiomyopathy nonspecific had characters of several cardiomyopathies.

**Hypertensive heart disease (n = 8) and left ventricular hypertrophy without hypertension (n = 7).

Of all 86 patients, altogether 61 (71%) had left ventricular LGE present on CMR. The median LGE extent in all patients was 7% (0 – 25%) of LV volume and in LGE positive patients alone 13% (6 – 32%). Altogether 56 patients (65%) had SWMA, with a median SWMA score 4 (0 – 12) in all patients and 7 (4 – 19) in SWMA positive patients. In all 1462 segments (17 segments/patient x 86 patients) the extent of LGE and SWMA were significantly associated (p < 0.001). Of all segments, 403 (28%) had LGE and 472 (32%) SWMA. Abnormal motion was found in 20% of segments without enhancement and in 75% of segments enhancing more than 50%.

Baseline clinical characteristics and imaging parameters of all study patients and patients discriminated with the presence of LGE and SWMA are shown in Tables 2 and 3. At baseline, the presence of LGE (vs. absence of LGE) and SWMA (vs. absence of SWMA) were significantly associated with NYHA-class, CHF and LVEF. Both LGE positive (vs. LGE negative) and SWMA positive (vs. SWMA negative) patients had significantly higher NYHA-class (II vs. I, p = 0.001 for both), more frequent CHF (56% vs. 16%, p = 0.002; 64% vs. 7%, p < 0.001) and decreased median LVEF on CMR (45% vs. 60%, p < 0.001; 42% vs. 65%, p < 0.001). At baseline, there were no significant differences associated with the presence of LGE (vs. absence of LGE), or SWMA (vs. absence of SWMA), in myocardial injury biomarkers, arrhythmias or conducting abnormalities.

**Table 2:** **Baseline clinical characteristics of all patients, and patients discriminated with the presence of late gadolinium enhancement (LGE) and segmental wall motion abnormality (SWMA)**

	All patients (n = 86)	LGE negative (n = 25)	LGE positive (n = 61)	p-value	SWMA negative (n = 30)	SWMA positive (n = 56)	p-value
**Demographics**							
Age, year	53 (42–61)	49 (42–59)	53 (42–63)	0.278	51 (42–59)	53 (42–62)	0.336
Gender, female	39 (45)	15 (60)	24 (39)	0.131	20 (67)	19 (34)	0.007
**Cardiovascular risk factors**							
Dyslipidemia	51 (59)	14 (56)	37 (61)	0.871	17 (57)	34 (61)	0.893
Hypertension	34 (40)	7 (28)	27 (44)	0.247	10 (33)	24 (43)	0.529
Diabetes	7 (8)	2 (8)	5 (8)	1.000	0 (0)	7 (13)	0.108
Smoking	19 (22)	6 (24)	13 (21)	1.000	6 (20)	13 (23)	0.944
Family risk for CAD	11 (13)	5 (20)	6 (10)	0.285	5 (17)	6 (11)	0.504
Sum of risk factors	1 (1–2)	1 (0–2)	1 (1–2)	0.513	1 (1–2)	1 (1–2)	0.406
**Symptoms**							
Syncope or presyncope	21 (24)	9 (36)	12 (20)	0.185	9 (30)	12 (21)	0.536
Palpitation	34 (40)	13 (52)	21 (34)	0.204	14 (47)	20 (36)	0.448
Chest pain	31 (36)	9 (36)	22 (36)	1.000	12 (40)	19 (34)	0.746
NYHA-class	2 (1–3)	1 (1–2)	2 (1–4)	0.001	1 (1–2)	2 (1–4)	0.001
**Congestive heart failure**	38 (44)	4 (16)	34 (56)	0.002	2(7)	36 (64)	< 0.001
**Arrhythmias**							
Atrial fibrillation	25 (29)	6 (24)	19 (31)	0.688	5 (17)	20 (36)	0.109
Ventricular fibrillation	9 (10)	3 (12)	6 (10)	0.715	3 (10)	6 (11)	1.000
Sustained VT	9 (10)	1 (4)	8 (13)	0.274	1 (3)	8 (14)	0.152
Nonsustained VT	16 (19)	6 (24)	10 (16)	0.542	5 (17)	11 (20)	0.962
**Conducting abnormalities**							
AVB of any grade	39 (45)	7 (28)	32 (53)	0.067	9 (30)	30 (54)	0.062
Distal AVB	7 (8)	3 (12)	4 (7)	0.409	3 (10)	4 (7)	0.691
Cardiac enzyme elevation*	32 (37)	8 (32)	24 (39)	0.693	9 (30)	23 (41)	0.436

Values are median (IQR) or n (%).

*Troponin T, Troponin I or CK-mb.

*Abbreviations:* *AVB* atrioventricular block, *CAD* coronary artery disease, *IQR* interquartile range, *LGE* late gadolinium enhancement, *NYHA-class* New York Heart Association classification of functional capacity, *SWMA* segmental wall motion abnormality, *VT* ventricular tachycardia.

**Table 3:** **Baseline imaging parameters of all patients, and patients discriminated with the presence of late gadolinium enhancement (LGE) and segmental wall motion abnormality (SWMA)**

	All patients (n = 86)	LGE negative (n = 25)	LGE positive (n = 61)	p-value	SWMA negative (n = 30)	SWMA positive (n = 56)	p-value
**Echocardiography**							
LVEDD, mm	55 (48–61)	48 (44–54)	56 (50–63)	< 0.001	48 (44–54)	59 (53–67)	< 0.001
LVEF, %	50 (33–62)	62 (57–71)	42 (25–56)	< 0.001	63 (60–70)	38 (23–52)	< 0.001
**CMR**							
LVEDV, ml/m2	78 (64–110)	71 (60–82)	87 (66–111)	0.050	68 (60–76)	97 (69–120)	< 0.001
LVESV, ml/m2	36 (25–69)	26 (21–32)	46 (30–73)	0.001	25 (20–31)	55 (35–85)	< 0.001
SV, ml	73 (58–85)	75 (65–89)	72 (55–84)	0.227	76 (65–82)	70 (52–88)	0.291
LVEF, %	52 (35–61)	60 (56–69)	45 (32–58)	< 0.001	65 (59–69)	42 (30–53)	< 0.001
LGE presence	61 (71)	-	-	-	9 (30)	52 (93)	< 0.001
LGE extent, score	5 (0–17)	-	9 (4–22)	-	0 (0–2)	11 (4–23)	< 0.001
LGE extent, % LV volume	7 (0–25)	-	13 (6–32)	-	0 (0–3)	16 (6–34)	< 0.001
SWMA, presence	56 (65)	4 (16)	52 (85)	< 0.001	-	-	-
SWMA extent, score	4 (0–12)	0 (0–0)	5 (3–17)	< 0.001	-	7 (4–19)	-

Values are median (IQR) or n (%).

*Abbreviations:* *CMR* cardiovascular magnetic resonance, *IQR* interquartile range, *LV* left ventricular, *LVEF* left ventricular ejection fraction, *LGE* late gadolinium enhancement, *LVEDD* left ventricle end-diastolic diameter, *LVEDV* left ventricle end-diastolic volume, *LVESV* left ventricle end-systolic volume, *SV* stroke volume, *SWMA* segmental wall motion abnormality.

### Follow-up

After CMR, patients were followed-up for MACE in median 835 (IQR: 780 – 998) days. Of 86 patients, altogether 15 (17%, annual event rate: 7.6%/year) reached an endpoint during follow-up: 5 cardiovascular deaths, 2 cardiac transplantations and 8 aborted sudden deaths.

Univariate Cox regression analysis was performed to find significant unadjusted predictors of MACE during follow-up, see Table 4. Significant predictors of adverse outcome were NYHA-class III-IV (hazard ratio [HR] 2.8, p = 0.049), sustained VT (HR 3.8, p = 0.023), atrioventricular block of any degree (HR 3.8, p = 0.022), stroke volume (HR 0.968, p = 0.032), LVEF on CMR (HR 0.959 per 1% increase in LVEF, p = 0.009), LGE volume (HR 1.028 per 1% increase in LGE, p < 0.001) and SWMA score (HR 1.067 per 1 point increase in SWMA, p = 0.012).

**Table 4:** **Univariate Cox regression analysis of event-free survival**

	HR	95% CI	p-value
**Demographics**			
Age, year	1.020	0.980 - 1.060	0.333
Gender, female	1.094	0.397 - 3.016	0.863
**Symptoms**			
Syncope or presyncope	1.093	0.348 - 3.434	0.879
Palpitation	1.369	0.497 - 3.777	0.544
Chest pain	0.837	0.286 - 2.449	0.745
NYHA-class (III - IV)	2.820	1.003 - 7.928	0.049
**Congestive heart failure**	2.704	0.924 - 7.913	0.069
**Arrhythmias**			
Atrial fibrillation	2.256	0.818 - 6.223	0.116
Ventricular fibrillation	0.559	0.073 - 4.250	0.574
Sustained VT	3.807	1.207 - 12.013	0.023
Nonsustained VT	1.122	0.317 - 3.975	0.859
**Conducting abnormalities**			
AVB of any grade	3.801	1.210 - 11.945	0.022
Distal AVB	0.758	0.100 - 5.764	0.789
Cardiac enzyme elevation*	1.540	0.558 - 4.247	0.404
**CMR**			
LVEDV, ml/m2	1.004	0.991 - 1.016	0.556
LVESV, ml/m2	1.008	0.996 - 1.020	0.213
SV, ml	0.968	0.940 - 0.997	0.032
LVEF, %	0.959	0.930 - 0.990	0.009
LVEF < 50%	3.813	1.213 -11.981	0.022
LGE, presence	6.329	0.832 - 48.143	0.075
LGE, extent, score	1.042	1.019 - 1.065	< 0.001
LGE, extent, % LV volume	1.028	1.013 - 1.044	< 0.001
SWMA, presence**			0.002
SWMA, extent, score	1.067	1.014 - 1.122	0.012

*Troponin T, Troponin I or CK-mb.

**HR was not calculated for the presence of SWMA, since there were no events in the group of patients without SWMA; Univariate p-value was walculated with Log rank test.

*Abbreviations:* *AVB* atrioventricular block, *CAD* coronary artery disease, *CI* confidence interval, *CMR* cardiovascular magnetic resonance, *HR* hazard ratio, *LV* left ventricular, *LVEF* left ventricular ejection fraction, *LGE* late gadolinium enhancement, *LVEDD* left ventricle end-diastolic diameter, *LVEDV* left ventricle end-diastolic volume, *LVESV* left ventricle end-systolic volume, *NYHA-class* New York Heart Association classification of functional capacity, *SV* stroke volume, *SWMA* segmental wall motion abnormality, *VT* ventricular tachycardia.

Considering the presence of LGE, there was an event rate of 26% (14 of 61) in LGE positive patients compared with 4% (1 of 25) in LGE negative patients (p = 0.041, Log rank), showing also a trend toward significance in Cox regression (HR 6.3, p = 0.075). Correspondingly, the presence of SWMA was a significant predictor of worse outcome during follow-up (p = 0.002, Log rank), but the hazard ratio was not calculated since there were no events in the group of patients without SWMA.

Multivariate analysis was performed to find the adjusted predictors of MACE, see Table 5. The best overall model to predict cardiac events included LGE volume (HR 1.027, p = 0.003), sustained VT (HR 4.8, p = 0.011) and LVEF (HR 0.962, p = 0.034).

**Figure 1:**
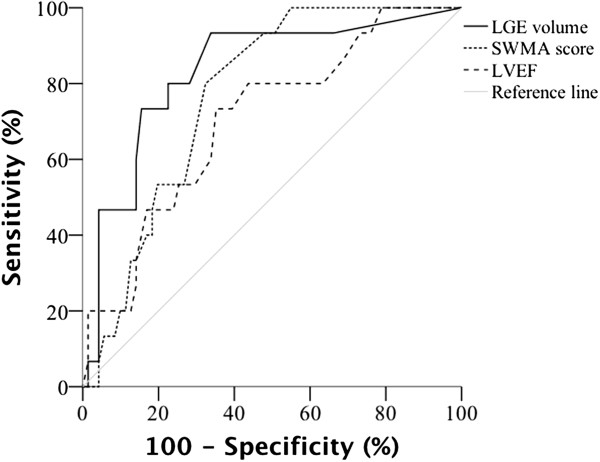
**Receiver operating characteristic curves of late gadolinium enhancement (LGE) volume, segmental wall motion abnormality (SWMA) score and left ventricular ejection fraction (LVEF) for prediction of events during follow-up, with corresponding area under curve 0.832 (95% confidence interval [CI]: 0.716 – 0.948), 0.769 (95% CI: 0.666 – 0.872) and 0.704 (95% CI: 0.565 – 0.844).** In the patient cohort of suspected nonischemic cardiomyopathy with relatively high prevalence of LGE and SWMA (71% and 65% of patients) at baseline, the optimal cut-off values with the best combination of sensitivity and specificity were LGE volume of ≥ 17%, SWMA score of ≥ 5 and LVEF < 46%.

ROC curves of LGE volume, SWMA score and LVEF on CMR were analysed to find optimal cut-off values for prediction of events during follow-up, with corresponding area under curve (AUC) 0.832 (95% confidence interval [CI]: 0.716 – 0.948), 0.769 (95% CI: 0.666 – 0.872) and 0.704 (95% CI: 0.565 – 0.844), see Figure 1. The cut-off value with the best combination of sensitivity and specificity for LGE volume was ≥ 17% (sensitivity: 80%; specificity: 78%), for SWMA extent score of ≥ 5 (sensitivity: 80%; specificity: 68%) and for LVEF the percentage of < 46% (sensitivity 73%; specificity: 65%).

**Figure 2:**
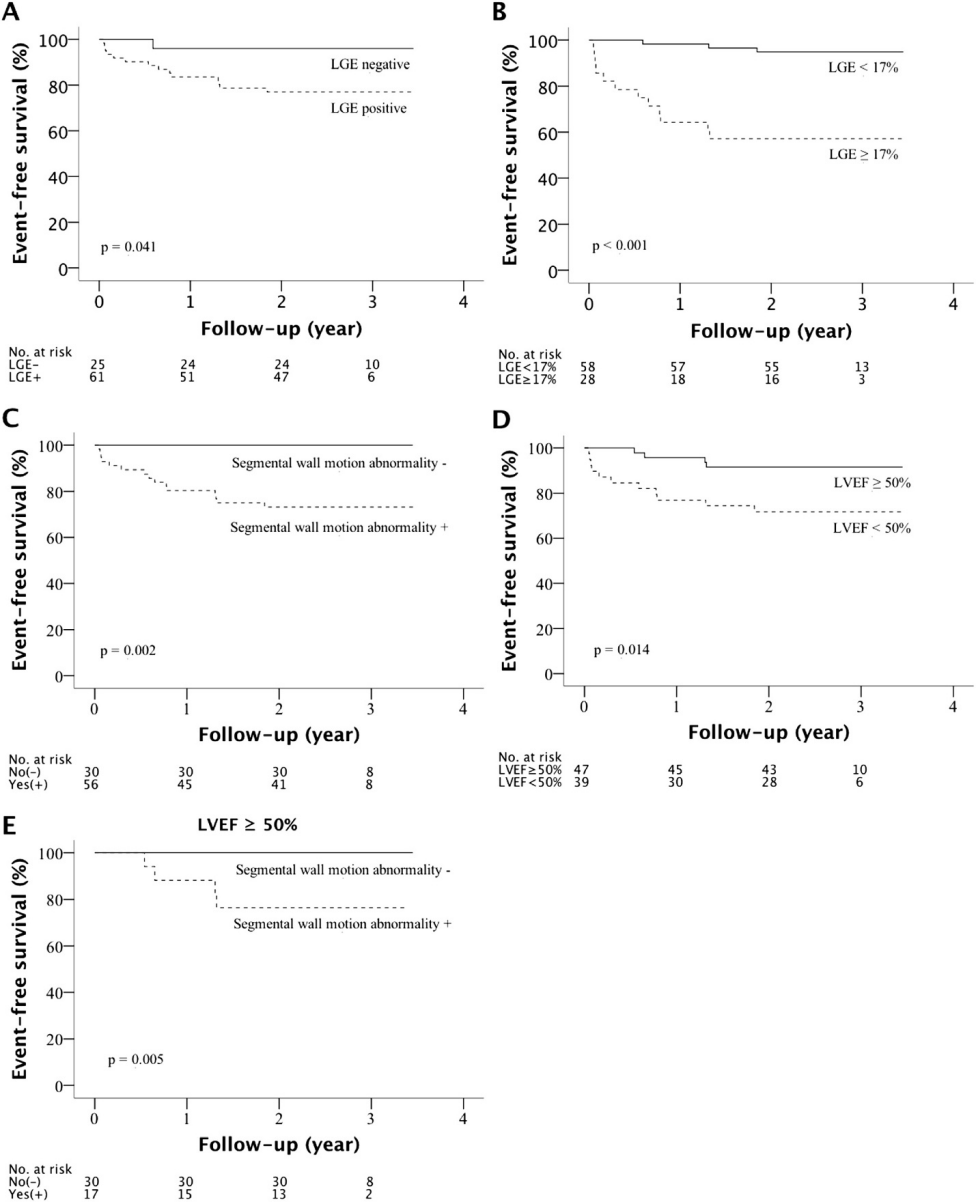
**Kaplan Meier analysis of event-free survival during follow-up in patients discriminated with the presence of late gadolinium enhancement (LGE) on cardiovascular magnetic resonance (CMR) (A), with LGE volume of ≥ 17% (B), with the presence of segmental wall motion abnormality (SWMA) (C), and with left ventricular ejection fraction (LVEF) of < 50% (D).** In the patient cohort with preserved LVEF (≥50%), the absence of SWMA (30 patients) resulted in no events during follow-up, while 17 patients with SWMA were still at risk with 4 events **(E)**.

Kaplan Meier curves of event-free survival of patients discriminated with the presence of LGE (Log rank, p = 0.041) and with LGE volume ≥ 17% (Log rank, p < 0.001) demonstrate that there was a high risk group of 28 patients (LGE volume ≥ 17%) with 12 events and a cumulative event ratio of 43% after three years (Figure 2A and B). Patients with SWMA (p = 0.002, Log rank) and patients with LVEF < 50% (p = 0.014, Log rank) were also at increased risk (Figure 2C and D). Furthermore, in the patient cohort with preserved LVEF (≥50%, 47 patients), the absence of SWMA (30 patients) resulted in no events during follow-up, but 17 patients with SWMA were still at risk with 4 events (Log Rank, p = 0.005) (Figure 2E).

**Table 5:** **Multivariate Cox regression analysis of event-free survival**

	HR	95% CI	p-value
**Model** (forward stepwise)			
LGE extent, % LV volume	1.027	1.009 - 1.044	0.003
Sustained VT	4.793	1.428 - 16.087	0.011
LVEF (CMR), %	0.962	0.929 - 0.997	0.034

*Abbreviations:* *CI* confidence interval, *HR* hazard ratio, *LGE* late gadolinium enhancement, *LV* left ventricular, *LVEF* left ventricular ejection fraction, *VT* ventricular tachycardia.

## Discussion

This study shows that in patients with suspected NICM, mainly presenting with ventricular arrhythmias or CHF, the extent of LGE on CMR independently predicts MACE compared to traditional risk factors. Even though the final diagnosis is uncertain, extensive amount of LGE should be considered as a sign of poor prognosis and activate more intensive diagnostics and surveillance. Furthermore, the absence of SWMA on CMR was a strong predictor of good prognosis with no cardiac events during follow-up, giving additional prognostic information also in the subgroup of patients with preserved LVEF (≥50%).

### Prognostic value of LGE in NICM

In our study, for every 1% increase in LGE volume, the risk of reaching MACE during follow-up increased 2.7%, independently of sustained VT and LVEF. The highest event rate was observed in the patients with LGE volume of ≥ 17%, with a cumulative event ratio of up to 43%.

In earlier studies, the prognostic value of LGE has been documented in several disease entities of NICM. In DCM, the presence and extent of LGE, typically mid-wall replacement fibrosis, has been shown to provide independent and incremental prognostic information [12, 13, 17]. In hypertrophic cardiomyopathy (HCM), the presence and extent of LGE predict adverse outcome [10, 11] and lately in a systematic meta-analysis of 1,063 patients with HCM, during 3.1-year follow-up, the presence of LGE was associated with cardiac death and all-cause mortality [26]. In suspected cardiac amyloidosis a characteristic circumferential endomyocardial LGE is a stronger predictor of mortality compared to other noninvasive parameters [15], and in suspected cardiac sarcoidosis the presence of LGE was the best adjusted predictor of adverse cardiac events [27]. Also, the presence of LGE has been shown to be the best independent predictor of mortality in biopsy-proven viral myocarditis compared to traditional cardiac signs and symptoms [28].

However, there is limited information on the prognostic value of LGE in nonselected consecutive patients with suspected or newly diagnosed NICM, reflecting the common clinical setting, and whether the quantification of LGE provides additional prognostic information in these patients. In a recent study of patients with newly diagnosed NICM, the presence of LGE was associated with worse prognosis, although only traditional risk markers, such as LV performance and cardiac biomarkers, were independent prognostic factors [8]. Our study adds to previous studies, that in clearly symptomatic patients with suspected NICM, the extent of LGE, along with its presence, carries prognostic value.

Of our study cohort, 76% of patients were eventually diagnosed with NICM, including 27% of patients with inflammatory cardiomyopathy. The histological basis for LGE in this sample was probably heterogenic, including replacement fibrosis, necrosis, oedema or amyloid infiltration. Patients with inflammatory cardiomyopathy may have wide-spread amounts of less intense LGE compared to myocardial infarction [29] *.* In this study, the optimal cut-off value for LGE extent for event prediction during follow-up was a volume of ≥ 17%. This is higher compared to a recent cohort study of patients with nonischemic DCM with an indication for ICD, where the optimal cut-off value of LGE extent was 6.1% for event prediction [16]. This difference in cut-off values is probably explained by different patient cohorts; our study did not include just patients with DCM but patients with suspected NICM, of which 27% had finally inflammatory cardiomyopathy having higher LGE volume in LGE positive patients (mean 22% [median 13%]) compared to DCM patients (mean 9%) in the other study. This demonstrates the importance of taking into account the reference patient population, while choosing the optimal cut-off value for LGE in risk prediction. Also, importantly, after the etiology of NICM is diagnosed, LGE volume should be interpreted in the context of that disease.

The HR of 1.027 associated with LGE extent in predicting MACE was smaller compared to recent studies of DCM (HR = 1.11 or HR = 1.16) [16, 17], but similar to a recent study of 217 consecutive HCM patients, in which LGE extent was associated with HR 1.15 for each 5% increase in LGE volume [10]. The mean LGE extent (in LGE positive patients) was similar between our study patients and HCM study patients (22% vs. 15.5%), but higher compared to DCM study patients (median 2.5% in the other, mean 9% in the other). Hence, in the sample of patients with relatively large amounts of LGE, a very small increase in LGE extent does not necessarily cause clinically meaningful increase in risk, although statistically significant.

### Prognostic value of segmental wall motion abnormality

In this study SWMA on CMR was present in 65% of patients. The unadjusted SWMA score predicted MACE during follow-up (1 point increase in SWMA score was associated with 6.7% increase in risk), but not independently of traditional prognostic factors such as LVEF. It is natural that SWMA score and LVEF have strong interrelation, since they both are measures of global LV function [24]. However, the absence of SWMA was a strong predictor of good prognosis in our patients. Furthermore, SWMA may also give prognostic information beyond preserved LVEF (≥50%), since in this subgroup patients with SWMA were still at risk for further events, although data was small.

### Study limitations

This study employs an observational follow-up study design. The number of patients enrolled to the study and who eventually reached MACE during follow-up was limited. However, all endpoints were life-threatening events. The patient cohort with suspected NICM at presentation was heterogenic in final diagnoses. Thus, the results of this study should not be interpreted in specific cardiac disease entities. However, patients with suspected NICM reflect the real-life setting in which the need of CMR is considered. In this study we did not evaluate the additional prognostic value of LGE or SWMA in relation to final diagnoses, although it is known that the etiology of NICM has influence in outcome [3]. Also, it must be reminded that LGE visualizes only myocardial enhancement in relation to “normal” myocardium and has limited ability to detect diffuse myocardial changes. Recently introduced extracellular volume quantification method based on gadolinium-enhanced CMR and myocardial T1-mapping seems to be useful in detecting diffuse myocardial fibrosis or homogeneously distributed infiltration, seen in many forms of NICM, thus potentially providing further prognostic information [30]. Finally, the presented cut-off values for LGE extent in this study represent only our patient sample, demonstrating the effect of increasing LGE on patient outcomes, and should be tested in independent study population.

## Conclusions

In suspected NICM, presenting with ventricular arrhythmias or heart failure, LGE extent gives additional prognostic information compared to traditional risk factors, while the absence of segmental wall motion abnormality may give prognostic information beyond normal LVEF. Even though the final diagnosis is uncertain in NICM, extensive amount of LGE should be considered as a sign of poor prognosis and activate more intensive diagnostics and surveillance.
